# Contemplation: Its Cultivation and Culmination Through the Buddhist Glasses

**DOI:** 10.3389/fpsyg.2021.800281

**Published:** 2022-04-05

**Authors:** Madhumita Chattopadhyay

**Affiliations:** ^1^Department of Philosophy, Jadavpur University, Kolkata, India; ^2^Centre for Buddhist Studies, Department of Philosophy, Jadavpur University, Kolkata, India

**Keywords:** *dhyāna citta*, *magga citta*, *bhūmi*, *samatha*, *vipassanā*, *brahmavihāra*, *śrāvaka*, bodhisattva

## Abstract

Buddhist account of consciousness provides a new way of looking into contemplation, where absorption into meditation does not only bring in changes in the neural level but in the very personality of the individual, turning him into a good human being. The Buddhists recommend the practice of *vipassanā*, literally meaning insight but actually standing for the realization of the supreme enlightenment breaking off all the internal fetters through the practice of seven different types of purity, such as purity of morals, mind, views, and insight, etc. In early and later Buddhist literature, it has been categorically emphasized that one should not practice solitary mindfulness but should practice enriching the higher qualities of the mind such as compassion, friendliness, etc. The point is that the contemplated individual, the Bodhisattva, should possess the perfection of wisdom (*prajñā-pāramitā*) and be equipped with the skill of *upāya*s. The practice of these *upāya*s, however, will not create any new bondage for the contemplative mind. When the individual is able to attain this broad outlook, he will be said to achieve the highest contemplation – this is the ultimate objective of Buddhist meditation. Meditation is needed for this change of outlook – transition from “I” to “we”. This is the only way to bring peace of mind and peace to the whole world.

## Introduction

### Difference in Ordinary and Philosophical Understandings of *Yoga*

In recent years, the term “meditation” or more correctly “*yoga*” has become very popular throughout the world, especially among health-conscious people. This popular usage makes the term “*yoga*” refer to the practice of certain bodily postures (*āsana*s), certain breath-control (*prāṇāyāma*) exercises, and practice of some forms of meditation such as reciting the word “*oṃ*”. Obviously, such so-called *yogic* practices have as their main aim removal of diseases through maintaining the fitness of the body. In other words, the ordinary understanding of *yoga* is body-oriented. However, this popular sense was not what was originally associated with the term “*yoga*” in Indian philosophy. There, it stood for a method of self-regulation aiming at the attainment of liberation (Gokhale, 2020).

Liberation (Bronkhorst, 1993)^1^ has been described as a stage free from all sorts of suffering. From the psychological point of view, it may be regarded as a state of mind, free from all sorts of tension, sorrows, and sufferings. Naturally, such a stage is desirable by all. In modern psychological terminology, it may be regarded as the stage of well-being. Generally, meditation or contemplation (*dhyāna* or *samādhi*) is considered to be its core means, retaining the other features such as bodily postures, breath-regulation, etc. in the periphery. This meditation or contemplation stood for the concentration of the mind at its optimum for the orthodox thinkers who believed that the ascetics or the saints alone are the only eligible persons and not the ordinary people with the fleeting mind. Buddha, however, did not accept such a view. With a socialistic and equalitarian outlook, he wanted to bring liberation within the reach of the common people, and that too not by lowering the standard of liberation, but by uplifting the consciousness of the common people to such a level that they can start the process of meditation to become a holy man or a saint (*ārya*) or a noble one to attain the stage of liberation.

### Definition of an Ordinary Person

An ordinary person, in the language of the Buddhists, a *pŗthagjana* (*puthujana* in Pāli), is the one who has not entered the path of seeing (*darśanamārga*) the four noble truths (*āryasatya*) or the supramundane noble factors (Nāṇamoli, 1991) (*āryadharma*). Ordinary persons, according to *Śrāvakabhūmi*, are of four varieties – (1) all the non-Buddhists; (2) those who are Buddhists in faith but whose spiritual faculties are weak; (3) those who are Buddhists and have keen spiritual faculties but whose roots of the wholesome factors are unripe; and (4) the bodhisattvas who wish to attain Bodhi awakening (that is awakening of the ultimate wisdom) not in the present life but in the future (Shukla, 1973). These four types of people can be broadly subdivided into two categories – that of ordinary people with complete bondage and that of adepts with partial bondage. Vasubandhu in his *Abhidharmakośabhāṣya* has regarded “non-possession of the noble paths” (Vasubandhu et al., 1998), as the characteristic of an ordinary person^2^. His condition is characterized as being entangled with nescience (*avidyā*) or ignorance (*ajǹāna*), delusion (*moha*), and different types of moral fault and defilements (*āsrava deşa* and *upakleśa*, etc.) which are responsible for his rebirth and repeated existence in this world full of pain and suffering. Nescience or ignorance stands for the traditional concept of a false view of self or belief in the self and also in adherence to a thing as of one’s own (*ātmātmīyābhiniveśa*). Out of this false view regarding the self, there arises attachment for worldly objects in them.

### Necessity of Meditation for Ordinary Persons: Buddhist Viewpoint

For such ordinary individuals, Buddha recommended contemplative practices which can remove their attachments for mundane phenomena and help them progress in their spiritual journey. With such practices, they ultimately become successful in attaining the final stage of contemplation when they will have a direct realization of the true nature of objects and become successful in attaining liberation. In Buddha’s teachings, there is an elaborate discussion of the different stages starting from the primary one, when the individual can start with the process of concentrating his mind on a fixed object. Another important feature of Buddha’s analysis of contemplation is that such practice not only removes ignorance step by step but also brings advancement or progress in our cognitive system. It also brings in a change in the culmination of our emotions which according to the Buddhists is as much needed for our well-being as is knowledge. Accordingly, the Buddhist literature, both the early and later forms, provides an elaborate analysis of the different stages of consciousness and also of the processes to move from one stage to another. In short, the Buddhist analysis provides a good example of contemplative practices, which if properly carried out, can lead to the cultivation of knowledge and of good emotion at the same time.

In terms of contemplative practice, the Abhidhamma literature maintains the primary role of the witness consciousness in the alleviation of human suffering. The meditative or contemplative practices recommended in this tradition for the attainment of the state of liberation or that of well-being are to a large extent systematic and scientific in nature. This scientific character is responsible for its popularity throughout the world for eliminating suffering even today when men are passing through difficult times in respect of their personal life and professional life. Modern psychological treatment also recommends meditation as a tool to get rid of anxieties, tensions, and depression, etc., and has a feel-good sensation. This meditation can be regarded as the modernized form of ancient contemplation. The advantages of meditation or contemplation have been stated in the text entitled *The Questions of King Milinda* thus. “Meditation preserves him who meditates it, it clears him from faults, it removes from him any bad reputation going him a good name, it destroys discount in him filling him with content, it releases him from all fear endowing him with confidence, it removes sloth for from him filling him with real, it takes away lust and ill-will and dullness, it puts an end to pride, it breaks down all doubt, it makes his heart to be at peace, it softness his mind, it makes him glad, it makes him grave, it gains him much advantage, it makes him worthy of reverence, it fills him with delight, it shows him the transitory nature of all compounded things, it puts an end to rebirth, and it obtains for him all the benefits of renunciation” (Rhys, 1890)^3^. The detailed analysis of the subtle changes taking place in consciousness or in our brain processes or our neurological network that has been worked out in the ancient Buddhist texts is lacking in the popular practice of meditation. As such to understand the necessity of meditation, and the interrelationship of meditation with well-being, and to know how the practice of meditation helps one to become a good social being and so is ultimately conducive to the betterment of society, it is essential to take a look into the Buddhist analysis of consciousness. In this paper, the aim is to present the Buddhist formulation of the steps to be followed to initiate the process of contemplation and proceed gradually from one stage to another to reach ultimately the state of well-being, where no stains of evil mental thoughts can be found.

### Consciousness: Its Analysis and Division From the *Abhidhamma* Point of View

The Buddhists apply their doctrine of causality or *pratītyasamutpāda* to find out the root cause of suffering. According to any causal principle, if A gives rise to B, then for the elimination of B, the cause of B, namely A, needs to be eliminated. Based on this principle, Buddha found that the main cause of suffering is ignorance which is there in the human mind from the beginning-less past. This ignorance proceeds from one birth to another through the flow of consciousness which leaves one body at the moment of death (*cūticitta*) and causes the beginning of another flow of consciousness which gets associated with a new body at the moment of conception and is known as the “new birth” (*paṭisandhi citta*); this flow of consciousness continues till the moment of death. Starting from the moment of conception to the moment of death flows the underlying subliminal state of consciousness (*bhavānga citta*) which often rises to the upper level of consciousness as a response to the stimulus received from outside. What we ordinarily regard as the mind along with its different functions is actually this upper form of consciousness.

Among the early Buddhist literature, the Abhidhamma (Vetter, 1988) texts provide us with a significant advanced model of consciousness. Depending on the nature of the objects toward which consciousness is directed, this mind or consciousness is regarded to be of two types – mundane and supramundane. The supramundane variety of consciousness has as its object *Nirvāṇa* or liberation, whereas the mundane variety of consciousness is directed toward mundane objects. Since *Nirvāṇa* or liberation is something very subtle and needs deep contemplation, people with an ordinary feeble mind cannot proceed to this stage without prior training. Accordingly, the Buddhists start with the process of training the mundane level of consciousness.

### Contemplative Consciousness: Its Beginning and Stages

The first stage of mundane consciousness is that of the ordinary one where the mind or the *citta* belongs to the sensual realms and moves from one sensual object to another very fast. It is actually from the second stage that the practice of contemplation begins. In fact, it is regarded as *dhyāna citta* (Gunaratna, 1980). This second stage of consciousness or mind is called *rūpāvacara citta*, where the mind or consciousness is being trained not to move from one object to another but to focus on one particular object. For a lay person, it is not easy to get the fleeting mind habituated with moving from one object to another, get concentrated on a fixed object. It needs long training. So, this stage of consciousness is thought to consist of five levels or stages. At the initial stage, the task is to draw the mind toward a definite object. Since ordinarily, the concentration is weak, the mind could fluctuate and the meditator could be subjected to frequent distractions. The sign may be lost due to any form of physical or mental disturbances and with it, the ability to recall the visual image sometimes becomes difficult. One-pointed, calm, and equanimous concentration on the learning sign is necessary and that subsequently leads to the “counterpart sign,” which is a far more vivid and clarified image than the previous one. This counterpart sign is not merely a clearer reproduction of the learning sign but is imbued with its own characteristic. With its arising the meditation attains access level concentration. At the access level, the frequency of distracting thoughts begins to decrease and the object of meditation starts to dominate his mental field; still, the possibility remains that intrusive sensory signals can divert the attention of the meditation from the object.

This second stage of consciousness consists of five constituents – *vitarka, vicāra, piti, sukha, and ekāggatā* – which can, respectively, be regarded as the initial application, sustained application, pleasure, happiness, and one-pointedness. This stage actually leads one to the ascent from the initial stage to the stage of absorbed meditation, through different intermediary stages. As one proceeds in the process of meditation gradually, one constituent part or factor of each stage lessens, except that in the fifth-stage happiness or *sukha*, gets replaced by equanimity, indifference, or *upekkhā*.

Let us take a brief look at the five constituent factors helping the individual proceed in the stage of absorption in meditation. “*Vitakka*” or initial application is the process by which the fleeting mind is driven toward a fixed or definite object. *Vicāra* or sustained application is the continuous exercise of consciousness to keep it fixed on that object and merged therein. *Piti* or pleasure is that constituent that elates the mind. Where there is pleasure, there is happiness or *sukha*. The difference between these two constituents is explained in the Abhidharma philosophy with the simile of a thirsty traveler who sees a transparent stream from a distance and the satisfaction that he gets after drinking the water. Here, his feelings excited by the sight of the stream is called pleasure, and the satisfaction that the individual gets after attaining the object is regarded as happiness (*sukha*). *Ekāggatā* or one-pointedness is that which deepens and develops into ecstatic meditation.

As one proceeds from one stage to another gradually, the constituents get dropped one after another, and ultimately, there remains one-pointedness and indifference which is the changed form of happiness. For example, in the initial stage, the *vitakka* or initial application is required as it was necessary to train the mind not to move from one object to another, but to get it fixed on any particular object. When the individual is successful in his practice of applying the mind to one object, rather than passing from one to another at a time, he is promoted from the first stage to the second one. In this second stage, then, there is no longer any need for the element of initial application. Accordingly, that element or constituent is dropped and what becomes important and necessary is the training for sustained application of the mind to the fixed object for a longer time. So, in the second stage, *vicāra* or sustained application of the mind is required. It helps the individual to concentrate on the object without “*vitakka*.” When one ascends to the third stage, one already has achieved success in attaining sustained concentration on an object. Therefore, there is no necessity for the first two factors or constituents; when they have dropped accordingly, they become defunct. In this stage, there exist only three factors, pleasure, happiness, and one-pointedness. When one attains the fourth stage, one enjoys the object by his sustained application of mind or concentration and so one gets the feeling of satisfaction which produces happiness. Accordingly, in the fourth stage of meditation, there remain only two factors operative which is happiness and one-pointedness. When one proceeds further and reaches the fifth stage, one becomes absorbed in the object completely. At this stage, the meditator is not at all concerned with any sort of feelings, happiness, or sorrow. He is rather engulfed with a kind of indifference. The mind is wholly absorbed in the object of meditation and becomes finer, subtler, and more tranquil than the former four. Absorbed in deep meditation, the mind is fully awake but remains undisturbed by objects and events happening outside, since the process of receiving data through sense faculties and transforming those data to be objects of knowledge is no longer operative. In other words, in spite of seeing, hearing, smelling, tasting, and touching, organs such as the eye, ear, nose, etc. are not able to see, hear, smell, taste, and touch their respective objects. The absolute concentration of the mind on the object of meditation paralyzes the function of the sense organs to give rise to sensory cognition which leads to attachment. On the contrary, the potentiality of the mind is increased to such an extent that it is capable of penetrating into the real nature of an object (Bannerjee and Chatterjee, 2018).

Attaining the final level of the second stage of contemplation, the progressive mind of the meditator does not want to remain contented with this attainment, rather feels a sort of dissatisfaction for the gross object of meditation. He has the feeling that the physical body is the source of all pain and suffering and because of this physical frame, there is no end to human suffering. Since the present object of meditation has similarity with the human body in having a physical frame, the contemplative mind gives up such coarse, gross things as his object of meditation and looks for something formless as the object for his contemplation. For this development of the mind, he ascends to the stage of formless contemplation, where the object of meditation is no longer anything gross but is subtle, formless. The first formless object that comes to the mind is that of space (*ākāśa*). Accordingly, the primary stage of this higher level of meditation starts with contemplating the infinity of space. Being absorbed in meditation on the notion of the infinity of space, the meditator finds his mind identified with infinity and no longer does the material object produce any effect on him in spite of the fact that his sense organs get related to the respective objects by the natural course of action. In the depth of his meditation, he feels his mind to be identified with infinity. The notion of duality ceases. The notion of oneness with the infinity of space brings in the concept of the infinity of consciousness. Generally, following the natural law of objects, consciousness or mind originates in one moment and dissolves in the next moment, giving rise to the idea of finite consciousness. But when through meditation, the meditator proceeds toward meditating on the infinity of consciousness, he appears to develop a feeling of indifference toward the idea of finite consciousness. His mind seems to transgress the limit of this state of finite consciousness and proceeds to the idea of infinite consciousness which is as if nothing and even the fraction thereof does not exist. In pursuance of the concepts of non-existence, there grows a feeling of nothingness – everything is void. This voidness or emptiness is manifested more and more. It is called “*akiñcana*,” that is, a nothingness that becomes the object of meditation. In this stage, the individual becomes more exalted and serene. Still, the individual wants to proceed further without being complacent. In the depth of serenity and calmness, consciousness along with all its faculties loses its grossness and acquires subtlety. Because of this subtleness, consciousness in this level cannot be regarded as perception (since it is no longer determinate); it cannot also be regarded as a case of non-perception (since here, consciousness is present, but because of its subtlety, it cannot be asserted). At this stage of contemplation, the level of consciousness is regarded to be such which is neither perception nor non-perception. We have seen that the individual through the practice of contemplation is able to attain that level of consciousness that is absorbed in the object of meditation and is able to enjoy tranquility and serenity. In spite of the fact that the mind is completely absorbed in contemplation, the object, however, subtle it may be, is something of the mundane nature. As he advances further, his mind or consciousness proceeds from the mundane to the supramundane one because the object turns out to become subtle from the gross, from the subtle to the subtler one, and no longer remains in the level of the mundane one and turns toward the supramundane to attain the concept of Bodhi.

### Supramundane Consciousness: Its Different Stages

When the focus of his mind has been elevated toward this supramundane he attains the stage of “*magga citta*” which will lead to *Nirvāṇa*. This *magga citta* is of four varieties – consciousness relating to the path of **stream-attainment** (*sotāpanna*), consciousness relating to the path of **once-returning** (*sakadāgāmī magga citta*), consciousness relating to the path of **never-returning** (*anāgāmī magga citta*), consciousness relating to the path of ***arhat***-**ship** (*arahatta magga citta*) (Nārada Thera, 2013).

The distinction of the four stages of supra-mundane consciousness is not based on the objects of contemplation but on the result, namely decrease in the number of rebirths, which actually indicates how much one is progressing in his journey toward the ultimate goal. One who plunges into this stream of contemplation cannot revert and is destined to attain his goal of Nirvāṇa in due course by maintaining steady progress. The progress of oneself in the journey depends on the degree of the removal of the bad roots (*mūla*) which stand as hindrances in his way and on the degree of the cultivation of the illimitable good qualities, which are technically known as *Brahmavihāra*. As such in the text, *The Questions of King Milinda*, the monk Nāgasena tells the king that “… the Tathāgatas, O king, long for the enjoyment of the bliss of attainment, of the joy of the tranquil state of Nirvana, that they devote themselves to meditation, with their minds fixed on the end they aim at” (Rhys, 1890)^4^.

The Buddhists admit ten fetters which are (1) dogma of the self (*satkāyadṛṣṭi*), (2) attachment to mere rules and rituals (*ṡīlavrataparāmarśa*), (3) doubt (*vicikicchā*), (4) excitement for sensuous pleasure (*kāmacchanda*), (5) hatred (*vyāpāda*), (6) lust for the world of forms (*rūparāga*), (7) lust for the formless world (*arūparāga*), (8) excitement (*auddhatya*), (9) conceit (*māna*), and (10) misconception (*avijjā*). Of these ten, the first five are called lower fetters as they belong to the world of sensual objects. The remaining five are regarded as upper fetters as they operate in the higher worlds of the form (*rūpa*) and formlessness (*arūpa*). In the formless stage, the lust for the world of form (*rūpaloka*) does not remain, but lust for formlessness or the *arūpaloka* and the other higher fetters may remain.

Contemplation is the initial stage of this supramundane consciousness. The misconceptions are removed there and all doubts are set at rest. Though the ego-sense and desire continue to exist, they lose their potentiality to hinder his upward journey and bind him down to the world of material objects. Because of the removal of misconceptions and doubts, the immoral consciousness that has its roots in misconception and doubts gradually get removed from his mind. Out of the twelve immoral qualities, five get eliminated as a result of contemplation in the initial stage of supramundane consciousness. Thus, the stream-entrant contemplator (*sotāpanna*) has been able to exhibit advancement in moral perfection, mental development, and insight. With this, the individual proceeds to a further step of advancement in respect of supramundane consciousness which is known as the *Sakadāgāmī* stage or the stage of once-returning. Attaining this stage of consciousness, all the desires regarding sensual material objects and also the ill-will associated with them gradually lose all their strength, as a consequence of which immoral consciousness rooted in greed and hatred become impotent to culminate into action. Further progress in following the eightfold noble path of moral perfection, mental development, and insight leads the individual to the third stage of contemplation which is known as the *anāgāmī magga citta* or stage of never-returning. This is called so because the practice of meditation in this stage leads to the complete removal of the two evils of sensual desire and ill-will. Consequently, the chance of returning to the world of sensual desires (*kāmaloka*) through rebirth gets completely stopped. In this advanced stage of contemplation, sensual desire and ill-will are completely removed, but other fetters, such as ego-sense, ignorance, etc. still remain, though they turn to be weak.

Continuous practice of the noble eightfold paths enables one to reach the fourth stage of supramundane consciousness, known as the *arhatta magga citta.* At this stage, the remaining fetters which had all lost their strength become very weak by constant contemplation and get completely removed. Just as at the advent of dawn, the darkness of the night is removed and the whole sky gets brightened by the golden rays of the morning sun; similarly, with the promotion of consciousness to the fourth stage, the mind shines in the full glory of the light of the supreme state. There remains no darkness, no impurity, and no ignorance. This unfettered free supramundane stage is called the *arhatta magga citta*. It is so-called because the individual has been able to “kill” (remove) all the “*ari*”-s, the enemies standing as a bar to the path of the attainment of the ultimate stage of liberation. In fact, attainment of this state is the goal of one’s spiritual development and the highest attainment of life. In Buddhism, this stage is also regarded as *nirodha samāpatti.*

### Process Consciousness and Resultant Consciousness

The *Abhidhamma* account of contemplation thus provides a description of the process and also the consequences which result from following the process of contemplation. In the Abhidhamma terminology, they are known as *magga citta* and the *phalacitta*, respectively. Through gradual practice of the different stages of supramundane consciousness one after the other, all the fetters or impurities get weakened; there arises immediately the respective resultant consciousness. The occurrence of the resultant consciousness from the practice of the process consciousness has often been compared with the lightening which flashes in between clouds for a second and then ceases. Contemplation starting from the second stage of mundane consciousness to the last stage of supramundane consciousness is called the “*jhāna citta vīthi*” or the contemplative process consciousness. It is also regarded as “*appana javana vīthi*.” The term “*appana*” stands for ecstatic contemplation where the mind or consciousness along with all the mental faculties is absorbed in the state of perfect *samādhi*-ecstasy. The question of clarity or indistinctiveness of an object does not arise, for ecstatic concentration cannot occur without the clarity of the object. However, identifying or registering the object as so and so does not happen in their contemplative procedure as the function of registering or identifying is operative in the case of consciousness of sensible objects (*kāmāvacara citta*) only. Thinkers of the Sarvāstivāda and Yogācāra schools describe the stage of practice in the following manner. After a certain number of preparatory steps such as *aśubhabhāvanā* “the realization of an impure body” which means the practice of meditating on the progressive deterioration of corpses, one enters the stage of *catvāri smŗtyupasthānāni*, where a practitioner first observes that the body (*kāya*) is impure (*aśuci*), that the sensations (*vedanā*) are suffering (*duḥkha*), that the mind (*citta*) is transient (*anitya*), and that the existent elements (*dharma*) are self-less (*anātman*), respectively, and then further observes in a unified way that the body, the sensation, the mind, and the existent elements are all impure, suffering, transient, and selfless. After this two-fold mindful observation which constitutes *mokşabhāgīya* together with the above mentioned preparatory steps such as *aśubhabhāvanā*, etc., the practitioner next enters into what is called *nirvedhabhāgīya* (*kuśalamūla*) stage (Funayama, 2011)^5^ which, according to Vinītadeva and also the Śravākayāna tradition, is such that the object of meditation is the four noble truths (*caturārya satya*). This process of *abhisamaya* or “full comprehension” comprises four stages, which may be described simply in the following manner.

First is the preparatory steps such as *aśubhabhāvanā* and *ānāpānasmŗtibhāvanā* followed by mindful observation or the establishment of mindfulness (*Smŗtyupasthāna*) leading to *uṣmagata* (the heated), then *mūrdham* (summit), and then *kṣānti* or acceptance leading to highest worldly elements. The condition of being an ordinary person (*pŗthagjana*) is retained through these stages. From the next moment starts the new phase as a holy being (*ārya*) which in the Śrāvakayāna tradition is regarded as the path of seeing (*darśanamārga*) and the path of contemplation (*bhāvanāmārga*) and in the case of Mahāyāna, as the Bodhisattva’s ten stages (*bhūmi*) (Funayama, 2011)^6^.

### The Notion of *Bhūmi*

The word “*bhūmi*” means field or jurisdiction. In the *Abhidharmakośabhāṣya*, it has been pointed out by Vasubandhu that field means the object or region of movement (*gativişaya*). Whatever be the object (or region) of movement of something is called its field. Those who have a great field are called “*mahābhūmika*” as they are present in all types of minds^7^. According to Vasubandhu, one-pointedness of mind or *ekāgratā* which is the same as *samādhi* is regarded as *mahābhūmika*, in the sense of having a great jurisdiction since all minds will have to be regarded as one-pointed. This is not so in another sense, because (in other states) one-pointedness may be weak^8^.

Florin^9^ (Florin, 2012), holds that *bhūmi* means foundation. He refers to Sthiramati’s interpretation as found in *Yogācārabhūmivyākhyā* that the *pañcavidhā yogabhūmi* refers to the five types of stages of spiritual practice due to their being the basis (*āṡraya*) and the ground (*adhiṣṭhāna*) of the cultivation (*bhāvanā*) of spiritual practice^10^.

The Buddhists hold that when a practitioner wants to attain a meditative stage of a higher plane or field (*bhūmi*), he has to develop detachment toward objects belonging to lower planes. This is regarded by Vasubandhu as *adhobhūmivairāgya*^11^. So, when the practitioner reaches the highest top, he has developed detachment toward all objects belonging to all the levels. Detachment is the conscious mastery over desire by one who is free from craving for empirical or mundane and also other-worldly or supramundane objects. It can be attained by one whose long *abhyāsa* or practice results in the accumulation of the wealth of merit and knowledge (Pradhan, 1950)^12^.

### Notions of *Samatha* and *Vipassanā*

Buddhist meditation is always associated with the two terms *samatha* and *vipassana. Samatha* is usually equated with contemplation or meditation (*samādhi*) and the purification of emotions. The *dhyāna citta* that we described earlier starting with the *rūpāvacara* and the *arūpāvacara citta*-s constitute the *samatha* practice. In short, the practice that aims to develop the concentration of the mind before the occurrence of insight is regarded as *samatha.* Depending on the personality of the individual, the practices of *samatha* vary. Some of the practices are not necessarily linked to a secluded practice. Others, such as the *kasiṇa*s and the *aśubha*s, require sustained attention that needs seclusion and tend to be practiced in a monastic context or on a period of extended meditation practice. Breathing mindfulness and body mindfulness, as parts of *samatha* practice, can be developed in daily life and also a meditative practice. Mindfulness of the body is said to lead to the start of meditation if pursued as a *samatha* practice whereas mindfulness of breath leads to the later four stages of meditation. The cultivation of the *brahmavihāra*s in activities in the world is encouraged, but they may be developed as a meditative practice also. That is, there is great variety and scope among the meditation subjects – each is assigned according to the temperamental needs of the mendicant and each is admitted in the canonical literature and followed in traditional practices. All the *samatha* practices are aimed at arousing the enlightenment factors and making the mind concentrated (*samāhita*), manageable (*kammanīya*), and purified (*pariśuddha*); accordingly, *samatha* is compared with the process of purification of gold which when purified and melted can be shaped in any way. The person practicing the mundane path attains a series of even deeper levels of serenity and increasingly attains a state of consciousness which is regarded to be a state of tranquility meditation known as *samatha*. This meditation or contemplation cannot, however, lead the individual to transcend the cycle of birth and death and attain the stage of liberation. *Samatha* is actually a method of contemplation by which the individual can get rid of evil emotions and stress of mind. *Vipassanā*, on the other hand, stands for the spiritual culture by minute observations of the activities of body and mind. It works on ignorance and volitional activities, through the way that the world is viewed and understood. The three facts of impermanence (*aniccatā*), the unpleasantness (*dukkhatā*), and non-self (*anattatā*) are being taken account of. The aim of *vipassanā*, therefore, is not the attainment of *rūpa*-meditation or *arūpa*-meditation, but the realization of the supreme *Nibbāna* removing all the internal fetters through the sevenfold purity like purity of morals (*ṡīla viśuddhi*), purity of mind (*citta viśuddhi*), purity of views (*diṭṭhi viśuddhi*), the purity of transcending doubt (*kaṇkhā-vitaraṇa-viśuddhi*), purity of vision in distinguishing between the right path and wrong path (*maggāmagga- ñānadassana-viśuddhi*), and the purity of insights (*ñāna-dassana-viśuddhi*). In some schools of Buddhism, the *samatha* is emphasized whereas, in some other *vipassanā*, practices are emphasized. In the canonical literature, we find evidence of both. Both these practices help the individual to eradicate all sorts of mental defilements and approach the attainment of *vijjā* (*vidyā*, knowledge), *magga-ñāna* (knowledge of the path leading to well-being) (Nandamālābhivaṃsa, 2003)^13^.

### Notion of *Bhāvanā*

In Buddhism, two paths are recognized for attaining the stage of liberation – the path of perception or realization, technically called *darśanamārga*, and the path of cultivation of the mind through meditative practices, technically known as *bhāvanāmārga*. The term “*bhāvanā*” means practice. This practice, according to the Buddhists, aims at (1) arousing or producing those good qualities which were not there, (ii) retaining those produced qualities without any failure also, (iii) increasing the qualities to a greater extent, and (iv) removing all the bad, immoral qualities present in the mind and taking steps for their non-occurrence in the future (Pradhan, 1950; Shaw, 2006)^14^. The path of realization enables one to realize that all the mundane objects are of the nature of suffering. Out of such realization, one takes steps to get rid of attachment. But to be successful in abandoning attachment, one has to follow the path of practice. According to the Buddhists, four defilements, namely attachment (*rāga*), hatred (*dveşa*), conceit (*māna*), and misconception (*avidyā*), stand as a hindrance to the attainment of well-being^15^. These hindrances can be overcome only through the repeated practice of meditation or contemplation^16^. As a result, of such practices arise detachment (*vairāgya*) toward objects. Progress in the practice of meditation has equal influence in the development of detachment toward objects as also in the occurrence of knowledge regarding the true nature of objects.

The later Buddhist thinkers hold that ignorant people wander in the cycle of rebirth because of their attachment toward worldly objects. The “*yogi*” or the person who meditates is able to overcome this delusion and by directing his great compassion toward all beings in the world, which proceeds to meditate further on *vipaśyanā* to realize the true nature of things. *Vipaśyanā* consists in the examination of the *bhūta*s or phenomena; *bhūta* stands for both *pudgala-nairātmya* and *dharma-nairātmya*, that is the notion of non-self and the notion of non-substantiality of everything. When this knowledge arises, the individual is able to realize the delusive nature of the concepts of “I,” “mine” and attains proficiency in removing all the dirt of mind. Proficiency in washing all the dirt or faults, he attains proficiency in meditating on *śūnyatā* for the removal of all illusory apprehension (*prapañca*). Hence, the *yogi*, with his eye of knowledge wide open, is able to overcome his *kleśa* foes with the weapon of *prajñā* and subsequently can wander about without fear, unlike a frightened coward with the eyes closed (Bhāvanākramaḥ of Ācārya Kamalaśīla, 2020)^17^.

### Later Buddhist Thought on the Cultivation of *Bhāvanā*

Later Buddhist works such as *Bhāvanākrama* have not only recommended the development of human consciousness to the optimum level so that all sorts of ignorance can be removed; they have also stressed finding out the factors that make our life miserable. The objective is to eliminate those factors and make human life happy and worth-living. Two root causes have been identified as responsible for human suffering – one is the past *karma* of the individual and the other is the disturbing emotions of love, hatred, and ignorance. Of the three, no doubt the latter is the most harmful one and dominates over the other two; it is responsible for the cycle of existence in this world and has simultaneous occurrence with consciousness itself. Generally, we love our friends and near ones and feel attached to them, out of the belief that they have done something good for us – they are our beneficiaries; on the other hand, we develop hatred and detachment toward our enemies out of the belief that they have done harm to us. This love for the friend and hatred for the enemy is very natural. However, the notions of friend and enemy are not fixed – the best friend today may turn out to be the worst enemy tomorrow; similarly, the much-hatred enemy may be found to be a benevolent friend later. Naturally, the emotions of love and hatred are not reliable; they are products of our narrow-minded attitude borne out of temporary and fleeting views of things and situations. If this narrow-mindedness can be transformed into a broader perspective with a more far-sighted attitude toward entities, futility of the hostile attitude and attachment will develop in the individual and this will give rise to the feeling of equanimity toward all. With the advent of the same feeling of equality toward friends, foes, and strangers, the individual will be regarded as successful in his contemplative practices. As he gradually progresses in this direction in meditation, he will extend his feeling of equanimity toward his neighbors, fellow beings gradually and ultimately toward all beings. At a certain point, all sentient beings in the world will be included as objects of his meditation. But to start with this attitude may be detrimental to his spiritual journey. Hence, the Buddhist masters of the past have advised the technic of gradually extending the scope of meditation from specific individuals to all beings.

Consequent to the cultivation of this feeling of equanimity toward all sentient beings, one can start meditating upon loving-kindness (*mettā bhāvanā*) which will help to develop the feeling of compassion in a swift and smooth manner. In the text *Bhāvanākrama*, it has been advised “Moisten the mental continuum with the water of loving-kindness and prepare it as you would a piece of fertile ground. When the seed of compassion is planted in such a mind, germination will be swift, proper, and complete. Once you have irrigated the mind-stream with loving-kindness, meditate on compassion” (Dalai, 2001)^18^. Meditation on compassion for a long time with great admiration will help to purify the mind-stream and get it ripened (Sharma, 2004). Then, the mind will get prepared for meditating on the perfect reality, just as it is possible to get fire by rubbing two pieces of wood only when the woods are dried. With such a purified mind, one will be able to get an extremely clear knowledge of phenomena free from conceptual elaboration^19^. Thus in the later, Bodhisattvayāna literature wisdom, and practice of compassion go hand in hand. It is believed that “the practice of compassion and the knowledge of emptiness will lead the individual to realize that the impurities of the mind can be removed and the state of omniscience can be achieved” (Dalai, 2001)^20^.

### Uniqueness of Buddhist Contemplation: Contemplation A Means to Well-Being for All

The Buddhists, starting from the earlier phase to the later one, believe that ordinary persons are eligible for attaining liberation. From the fact that the mega-encyclopedic text of spiritual journey, *Yogācārabhūmi*, (Asanga, 1957) consists of two parts *Śrāvakabhūmi* and *Bodhisattvabhūmi*, (Asanga and Nalinaksha, 1966) it seems that in the process of spiritual progress, a hierarchy of practice is admitted, namely first of following the *śrāvakayāna* path, then following the *pratyekabuddhayāna* path and then proceed to the course of spiritual cultivation as a Bodhisattva. Modern scholars like Florin think that if any form of hierarchy is to be admitted, that is not in terms of practice but in terms of religious ideals. From that point of view, *śrāvakayāna*, the lowest vehicle is to be placed first; this is followed by the path of the solitary Buddhas and finally the Mahāyāna. The *Śrāvakabhūmi* refers to three levels of practitioners (*yogācāra*) – beginners (*ādikarmika*), adepts (*kṛtaparicaya*), and practitioners who have transcended the practice of contemplation (*atikrāntamanaskāra*) (Florin, 2012)^21^. In other words, this text delineates the practices and the requirements of a contemplative, as he passes through the stages of a *śrāvaka, pratyekabuddha*, and Bodhisattva. The path of spiritual cultivation thus developed consists of two phases – one a preparatory phase which is more mundane in character and the other an advanced phase which is more of supramundane nature. When the practitioner first enters the preparatory stage, he is an ordinary person who is full of attachment, hatred, and other shortcomings. It is through undergoing a series of practices in restraint of morality (*śīlasaṃvara*), restraint of sense organs, moderate eating, mindful conduct, etc., that the novice is eligible to start with the process of meditation under the guidance of a master (Nyānaponika, 2017). It is after a thorough assessment of the psychic maturity and spiritual progression of the disciple that the preceptor chooses as the object of his meditation, any one of the following five – impurity (*aśubhā*), friendliness (*maitrī*), dependent origination (*idaṃ pratyayatāpratītyasamutpāda*), analysis of the elements (*dhātuprabheda*), and mindfulness of breathing (*ānāpānasmṛti*). Later, Mahāyāna thinkers emphasize two aspects of the gradual path leading to the attainment of the highest goal. The first one is acting for the benefit of others through the cultivation of virtues. This is the path of cultivating the qualities generally known as *pāramitā*s. The second one is related to the accumulation of gnosis or wisdom which will discern the nature of reality (*bhūtapratyavekṣā*). That is, for the Mahāyāna thinkers, both *dhyāna* and *prajñā* are important for reaching the state of ultimate well-being. In this respect, their view has been different from the earlier schools which one-sidedly championed for *prajñā* alone or for *dhyāna* over *prajñā.* The śrāvakayāna people put emphasis on the attainment of wisdom, without giving stress to compassion. Naturally, the goal aimed is *Nirvāṇa* for himself without thinking of the well-being of others. Later, Mahāyāna thinkers (Martin, 2002) or the Bodhisattvayāna thinkers recommend both cultivation of compassion and attainment of wisdom out of the belief that because of wisdom, these practitioners will be able to attain a stage where there is no chance of getting involved in mundane affairs. Again, because of the force of compassion, there is no chance of his staying on the stage of *Nirvāṇa*. Embracing the beings of the world without compassion is regarded to be bondage, and embracing all sentient beings with compassion is liberation. Thus, this later form of Buddhism provides a method of contemplation that leads to the accumulation of merit and wisdom with the determination to bind all sentient suffering beings of the world through the force of compassion. Being the epitome of wisdom and compassion, such practitioners aiming always for the welfare and happiness of other suffering beings will be the life-support for them. Thus, the essence of Buddhist contemplation (Wynne, 2007) is not to avoid the world, but to remain in this *saṃsāra* with a mind free from all sorts of attachment involving the thoughts of “I,” “mine,” etc. Such contemplation will widen his eyes to such an extent that he will no longer look for his own selfish pleasure and happiness, but will long for the happiness of all, well-being for all. The whole world will turn out to be his home and all mankind his brethren. Their suffering will be his own suffering, their happiness his happiness, and their well-being his own well-being. When the individual is able to attain this broad outlook, he will be said to achieve the highest contemplation – this is the ultimate objective of Buddhist meditation. Meditation is needed for this change of outlook – transition from “I” to “we”. This is the only way to bring peace of mind, peace to the whole world. Meditation or contemplation is the means to such well-being.

## Author Contributions

This manuscript tries to show following the original Buddhist texts available in Pali and Sanskrit, how the Buddhists provided an analysis of consciousness. In course of such analysis, they gave a quite novel account of how to progress in contemplation. The target group of this account was the ordinary people, the layman. The novelty of this analysis lies in the fact that through undergoing stages of such contemplation, the ordinary individual not only learns how to focus his attention on a particular object and get rid of all the factors that distract his attention and create different types of hindrances to disturb his mental peace and bring agony and discomfort. At the same time, it broadens his outlook so much that he feels for other sentient beings as much he feels for himself. The transition from the “I-consciousness’ to the “we-consciousness” is the uniqueness of Buddhist contemplation. This has been highlighted in this manuscript, which is not found in any other existing literature.

## Conflict of Interest

The author declares that the research was conducted in the absence of any commercial or financial relationships that could be construed as a potential conflict of interest.

## Publisher’s Note

All claims expressed in this article are solely those of the authors and do not necessarily represent those of their affiliated organizations, or those of the publisher, the editors and the reviewers. Any product that may be evaluated in this article, or claim that may be made by its manufacturer, is not guaranteed or endorsed by the publisher.

## Footnotes

1 Liberation has been expressed through different terminologies such as *mukti, apavarga, nirvāṇa, kaivalya*, and *mokṣa.* Different schools of Indian philosophy not only use different terminologies, and they also are not unanimous regarding the nature of this state. According to some, it is a state of profound happiness; according to others, it is a stage of supreme bliss; for some, it is a stage which cannot be described as either pleasure or pain, etc. However, in one respect all the schools are in agreement that this liberation is a stage which is completely free from all sorts of suffering.

2 *mārgasyāptiaptir işyate pŗthagjanatvam*/.*Abhidharmakośa* 2.40.

3 *The Questions of King Milinda*, p. 197.

4 *The Questions of King Milinda*, p.198.

5 Notion of this *nirvedhabhāgīya* stage has changed in the hands of later Buddhist thinkers like Kamalaúīla.

6 T. Funayama – “Kamalaúīla’s view on Yogic perception and the bodhisattva path”, p.99.

7 *Yo hi yasya gativiṣayaḥ sa tasya “bhūmiḥ” ityucyate/tatra mahatī bhūmireṣām iti mahābhūmikāḥ ye sarvatra cetasi bhabanti/Abhidharmakośabhāṣya* II.23.

8 *Abhidharmakośabhāṣya* VIII.1.

9 Florin, D – “Far from the Madding Strife for Hollow Pleasures: Meditation and Liberation in the *Śrāvakabhūmi*” pp 1-2 *Journal of the International College for Buddhist Studies* vol XVI, Year 2012, pp. 1–39.

10 Ibid, p.2.

11 *Abhidharmakośabhāṣya* VIII.14.

12 *Abhidharmakośabhāṣya* VII. 34, VIII.35, Asanga *Abhidharmasamuccaya* p. 96.

13 *Dve me bhikkhave dhammā vijjā bhāgiyā.* Quoted in *Samatha and Vipassanā: Concentration and insight meditation* by Ashin Dr. Nandamālābhivaṃsa, Dhammavijjālaya, Centre for Buddhist Studies, (English Version) Sagaing, 2003 p.21.

14 *Anuttpannānām kuśalānām dharmānām utpādāya yā bhāvanā*… *utpannānām kuśalānām dharmāòām sthitaya asaṃmoşāy bhūyobhāvāya vŗddhivipulatayai yā bhāvanā*… *utpannānām papakānām akuśalānām dharmānām prahānāya yā bhāvanā*… *anutpannānām pāpakānām akśakunām dharmānām anutpādāya yā bhāvanā*/*Abhidharmasamuccaya* pp 70–71.

15 *catvāro bhāvanāheyā tad yathā rāgah pratigtio māno vidyā ca. Abhidharmakośa* V.5.

16 *dīrghakālābhyāsah nirantarābhyāsah satkŗtyābhyāsaś ca Abhidharma kośabhāṣya* VII 34.

17 Tasmād visphārita-jñānacakṣuḥ prajnāúāstreṇa kleúārīn nirjitya nirbhayo viharan yogī, na tu kātarapuruṣa iva vinimīlitākṣaḥ/*Bhāvanākramaḥ* p.265.

18 *Stages of Meditation* p.54.

19 Tasyaivaṃ karuṇām upāyaṃ bodhicittaṃ ca satataṃ satkṛtya dīrghakālam abhyāsyataḥ krameṇa cittasantatiparipākād ativiśuddhakṣaṇotpādāt araṇimanthanodbhūtāgnivat bhūtārtha-bhāvanā-prakarṣa-paryanta-gamane sakala-kalpanā-jālarahitaṃ sphūṭataraṃ niṣprapañca-dharma-dhātvadhigamaṃ vimalaṃ niścala-nirvāta-dīpavat niścalaṃ pramāṇabhūtaṃ lokottarajñānaṃ sarvadharmanairātmya-svabhāva-tattva-sākṣātkāri darśana-mārgasaṃgṛhītaṃ paramārtha-bodhicittaṃ svarūpata utpadyate/*Bhāvanākramaḥ*, p.252.

20 *Stages of Meditation* p.154.

21 Florin – “Far from the Madding Strife” p.10.
